# Projected economic evaluation of the national implementation of a hypothetical HIV vaccination program among adolescents in South Africa, 2012

**DOI:** 10.1186/s12889-016-2959-3

**Published:** 2016-04-14

**Authors:** Nishila Moodley, Glenda Gray, Melanie Bertram

**Affiliations:** Perinatal HIV Research Unit, Faculty of Health Sciences University of the Witwatersrand, PO Box 114, Diepkloof 1864 Johannesburg, South Africa; South African HVTN AIDS Vaccine Early Stage Investigator Program (SHAPe), Seattle, WA United States; The South African Department of Science and Technology/National Research Foundation (DST/NRF) Centre of Excellence in Epidemiological Modelling and Analysis (SACEMA), University of Stellenbosch, Stellenbosch, South Africa; South African Medical Research Council, Tygerberg, South Africa; Vaccine and Infectious Disease Division, Fred Hutchinson Cancer Research Centre, Seattle, WA USA; Health Systems Governance and Finance, World Health Organization, Geneva, Switzerland

**Keywords:** HIV, Vaccine, Cost-effective, LYG, ICER, Antiretroviral therapy (ART)

## Abstract

**Background:**

Adolescents in South Africa are at high risk of acquiring HIV. The HIV vaccination of adolescents could reduce HIV incidence and mortality. The potential impact and cost-effectiveness of a national school-based HIV vaccination program among adolescents was determined.

**Method:**

The national HIV disease and cost burden was compared with (intervention) and without HIV vaccination (comparator) given to school-going adolescents using a semi-Markov model. Life table analysis was conducted to determine the impact of the intervention on life expectancy. Model inputs included measures of disease and cost burden and hypothetical assumptions of vaccine characteristics. The base-case HIV vaccine modelled cost at US$ 12 per dose; vaccine efficacy of 50 %; duration of protection of 10 years achieved at a coverage rate of 60 % and required annual boosters. Incremental cost-effectiveness ratios (ICER) were calculated using life years gained (LYG) serving as the outcome measure. Sensitivity analyses were conducted on the vaccine characteristics to assess parameter uncertainty.

**Results:**

The HIV vaccination model yielded an ICER of US$ 5 per LYG (95 % CI ZAR 2.77–11.61) compared with the comparator, which is considerably less than the national willingness-to-pay threshold of cost-effectiveness. This translated to an 11 % increase in per capita costs from US$ 80 to US$ 89. National implementation of this intervention could potentially result in an estimated cumulative gain of 23.6 million years of life (95 % CI 8.48–34.3 million years) among adolescents age 10–19 years that were vaccinated. The 10 year absolute risk reduction projected by vaccine implementation was 0.42 % for HIV incidence and 0.41 % for HIV mortality, with an increase in life expectancy noted across all age groups. The ICER was sensitive to the vaccine efficacy, coverage and vaccine pricing in the sensitivity analysis.

**Conclusions:**

A national HIV vaccination program would be cost-effective and would avert new HIV infections and decrease the mortality and morbidity associated with HIV disease. Decision makers would have to discern how these findings, derived from local data and reflective of the South African epidemic, can be integrated into the national long term health planning should a HIV vaccine become available.

## Background

South Africa has the largest human immunodeficiency virus (HIV) epidemic in the world [1]. In 2012, 6.4 million South Africans were living with HIV; 203,000 individuals had lost their lives to it and another 395,000 South Africans had acquired the infection [2, 3]. South Africa’s life expectancy was understandably adversely affected by the considerable burden of HIV disease [4]. However, life expectancy had since increased from 53 years in 2006 to 61 years in 2012, and ensuring its continued improvement remains a priority of the national department of health [5]. The gains made in improving life expectancy are in no small part attributable to ‘the largest antiretroviral (ART) rollout in the world’ that South Africa has managed to achieve [6]. To sustain this achievement is no mean feat. The growing number of patients previously initiated on ART need to be retained in care. While the public sector retention rate approximates 75 % after one year on treatment, South Africa needs to continuously enroll in excess of 500 000 new patients onto ART annually to maintain an ART enrolment ratio exceeding 1.3 [4]. This brings into question the long term sustainability of the ART program considering the massive financial and human resource implications the expansion of ART program entails [7].

Data suggests that close to 25 % of all new HIV infections occurred among young women aged 15–24 years, emphasizing this group as a major driver of the epidemic [2]. The HIV prevalence in this age group is important as it serves as a proxy for HIV incidence. HIV prevalence declined by 18 % in this age group from 2008 to 2012, from 8.7 % to 7.1 %, however there remains a need for intensified prevention efforts [8]. Despite massive accomplishments made in establishing the ART program, the women aged 15 – 24 years persist as the group with the poorest access to this life-saving treatment. The barriers that young people face in accessing public health services has been well documented [9]. Issues concerning lack of confidentiality and privacy, unfriendly and judgmental attitudes of health care staff and inaccessible clinic hours persist [10, 11]. It was against this backdrop that the re-engineering of primary health care in South Africa targeted the development of a school-based sexual and reproductive health service as a priority [12].

The current HIV prevention program has enjoyed limited success in tackling the high rate of new infections in South Africa, highlighting the need for an alternative intervention. Vaccines are regarded at the most cost-effective prevention intervention in the world [13]. Rerks-Ngam et al tested the first HIV vaccine regimen (RV144/Thai Trial) to show moderate vaccine efficacy in humans in Thailand (2009) [14]. The study evaluated a prime-boost strategy, priming with a recombinant canarypox vector (ALVAC-HIV[vCP1521]) administered at baseline, then at week 4, 12 and 24 with recombinant glycoprotein 120 subunit vaccine (AIDSVAX B/E) boosts given with the ALVAC at weeks 12 and 24.

The prime-boost HIV vaccine regimen used resulted in modest efficacy of 31 % over 3.5 years [14]. While the effects were not durable, they were indeed promising. After undergoing modifications to optimize the HIV vaccine regimen by making it Clade C specific and changing the protein and adjuvant, a potential vaccine regimen was entered into Phase I/IIb clinical trials at six major South African centers to assess safety and immunogenicity (HIV Vaccine Trial Network (HVTN) 100 study) [15]. Additionally, a pivotal phase IIb/III HIV vaccine efficacy trial is planned to take place in South Africa designated HVTN 702, which will evaluate the same regimen [as HVTN 100], should HVTN 100 prove to be immunogenic.

The aim of this analysis was to guide decision makers in assessing the value of national implementation of a potential HIV vaccine among school-based adolescents in South Africa. The work determined the impact of vaccination on HIV disease burden and associated health costs, and evaluated the cost-effectiveness and potential changes in life expectancy based on the premise that school-based care would address the issues of equity and accessibility in health care that adolescent South Africa faces.

## Methods

The study methodology was compliant with the reporting guidelines of the Consolidated Health Economic Evaluation Reporting Standards (CHEERS) statement [16].

### Study overview

Ten year old adolescents attending South African schools in 2012 were considered for vaccination. This intervention program was introduced as part of the national health initiative to introduce school-based sexual and reproductive health services [12], and targeted learners prior to the onset of sexual activity. The cohort was modelled through a lifetime horizon of 70 years inclusive, which exceeded the current estimated life expectancy of 60.6 years in South Africa [3]. The rationale for this was that life expectancy is rapidly changing in the South African environment and this cohort was considered to probably have a greater life expectancy. The assumption made was that the HIV vaccine would be incorporated into the South African Expanded Program of Immunization and would be administered at school level. The health service provider (provider) perspective was adopted as the information generated was intended to inform national health decision making. The hypothetical HIV vaccine was modelled as a prevention strategy that reduced the HIV disease burden and associated mortality. The vaccine strategies were considered against the system of HIV counselling and testing (HCT) and the national rollout of ART that constituted the standard of care (comparator model) in South Africa [17, 18]. The intervention model combined the current standard of care with the HIV vaccination strategy as both programs would be delivered simultaneously. A discount rate of 3 % was applied to the economic costs and health outcomes, as recommended by the World Health Organization CHOosing Interventions that are Cost-Effective (WHO-CHOICE) guidelines [19]. The epidemiology of South African epidemic is described in Table 1.

**Table 1:** South African population by age groups exploring ARV treatment access. The HIV epidemiology of South Africa is described. The treatment shortfall represents those eligible for ART but unable to access it

Age groups	Population	Susceptible	Prevalence^a^	On ARV treatment^a^	Treatment shortfall^b^
10–19	10 264 690	9 982 612	282 078	78 176	163 605
20–29	11 010 305	9 386 287	1 624 018	411 831	941 930
30–39	9 008 794	6 521 402	2 487 392	775 604	1 442 687
40–49	4 479 445	3 329 718	1 149 727	358 501	666 842
50–59	3 367 397	2 883 570	483 827	204 740	280 620
60+	3 665 571	3 534 983	130 588	55 260	75 741
Totals	41 796 202	35 638 572	6 157 630	1 884 112	3 571 425

^a^Shisana O et al. South African National HIV Prevalence, Incidence and Behaviour Survey, 2012. Cape Town, HSRC Press. 2014

^b^UN Joint Program on HIV/AIDS (UNAIDS). The Gap Report. 2014

### Outcome measures

Life years gained (LYG) was measured in terms of its impact on mortality. The LYG concept represents a modified mortality measure which considers remaining life expectancy. More weight is accrued to the life of a young child than an elderly person, because saving the life of a young child will accrue more life years than saving the life of an elderly person. The life years are calculated as the “remaining life expectancy at the point of each averted death” [20]. Life tables are generally setting specific or standardized for a geographic area. Using the information generated in these life tables, we are able to derive life expectancies for a specific population.

The HIV vaccine described for implementation was hypothetical as it is currently undergoing Phase I/II clinical trials. The HIV vaccine characteristics were determined by the target product profile formulated by the Pox-Protein Private Public partnership (P5), developed to build on the success of the RV144/Thai trial and evaluate potential HIV vaccine candidates to determine their public health impact [21]. The regimen included in this economic evaluation mirrored the ongoing HVN 100 study which adapted the ALVAC prime ALVAC/gp120/adjuvant boost of the RV144/Thai trial but added an additional ALVAC/gp120/adjuvant boost at month 12. This boost at month 12 was added to circumvent the waning of the immune response documented in the RV144/Thai trial a year after initial vaccine administration.

The estimated vaccination coverage was 60 % (range: 40–70 %). This represents a slight underestimation of the 68 % reported for coverage of the 3rd dose of diphtheria, tetanus and pertussis toxoid (DTP3) which has been validated as a proxy for national immunization performance [22]. The base-case HIV vaccine modelled cost US$ 12 per dose (range: US$ 2–24), had a vaccine efficacy of 50 % (range: 30–70 %) and the duration of protection of 10 years (achieved through the administration of annual boosters). The declining immunity reported in the RV144/Thai trial (particularly in the year following administration) reaffirmed the need for booster injections. Annual boosters may be far from pragmatic but merely represented an overestimation of costs in this evaluation. The vaccine price of US$ 12 was roughly based on the human papillomavirus (HPV) vaccine available on government tender at US$ 17. Markedly reduced vaccines prices deemed plausible given the strides made in negotiating lower priced ART medications and HPV vaccines in the public sector [23, 24]. Pooled utilities relating to HIV/AIDS (acquired immunodeficiency syndromes) were derived from a meta-analysis and were used for the cost-effectiveness analyses of HIV related interventions [25].

### Study inputs

Input parameters are shown in Table 2. Estimated vaccination coverage of 60 % of adolescents approximated 6 million individuals receiving the initial course. Delivery of health services was conducted at the schools. HIV related costs were estimated identified from the 2013 national HIV treatment guideline [18]. Patients would be consulted by primary health care (PHC) nurses and more complicated cases would be referred. Pharmaceutical costs included ART, treatment of sexually transmitted infections (STI) and condoms. In addition to the costs accumulated in the comparator group, the intervention included the vaccine and its delivery. Laboratory tests conducted by the National Health Services Laboratory, costing of medication, consumables and additional pharmaceuticals and valuations of medical personnel cost based on the Uniform Patient Fee Schedule (UPFS) were sourced from the National Department of Health. All costs were adjusted to the common year 2012. Costs were converted from South Africa rand (ZAR) to United States dollar (US$) using the average exchange rate for 2012, thus allowing for international comparison (US$ 1 = ZAR 8.21) [26]. HIV related disease transition probabilities were obtained from the South African literature and are shown in Table 3.

**Table 2:** Parameter costs and economic considerations. The estimates were obtained from relevant South African literature for the year 2012

HIV vaccine characteristics	Value	(Range)	Reference
Coverage	60 %	(40–70)	Assumption
Price (US$)	12	(2–24)	[47]
HIV vaccine efficacy	50 %	(30–70)	Assumption
Economics	Value	(Range)	Reference
Cost discount rate	3.0 %	(0–6 %)	[19]
Outcome discount rate	3.0 %	(0–6 %)	[19]
International comparison (ZAR: 1US$)	ZAR 8.21	-	[26]
HIV disease related costs	Distribution	Value	Reference
HIV prevention programme			
HIV vaccine	-	12	[21]
Vaccine delivery per dose	Gamma	17	[48–52]
Existing prevention programme (incl. HR)	Gamma	65	[49–53]
Voluntary counselling and testing (VCT) (per test)	Gamma	23	[49, 50]
Cost of HIV rapid testing	Gamma	2	[49, 50]
Current HIV programme (annual costs)			
Asymptomatic treatment (not on ART)	Gamma	131	[18, 53]
Symptomatic treatment (not on ART)	Gamma	137	[18, 53]
AIDS treatment (not on ART)	Gamma	182	[18, 53]
Patient on ART (average)	Gamma	424	[18, 23]
ART cost (annual)			
First-line regimen	Gamma	10	[23]
Second-line regimen	Gamma	27	[23]
Third-line regimen	Gamma	173	[23]
Laboratory costs (annual)			
First-line regimen (first year)	Gamma	17	[18, 53]
First-line regimen (subsequent years)	Gamma	46	[18, 53]
Second-line regimen	Gamma	46	[18, 53]
Third-line regimen	Gamma	92	[18, 53]
Not on ART	Gamma	65	[53]

**Table 3:** Disease transition probabilities showing annual progression risk. The possibility of transition from one HIV health state to the next is described. The estimates were obtained from relevant South African literature for the year 2012

Parameter	Distribution	Estimate	Reference
Change in HIV disease state			
Asymptomatic to symptomatic	Beta	0.32	[54]
Symptomatic to AIDS	Beta	0.20	[55]
AIDS to death	Beta	0.21	[54]
Change in drug regimens			
First-line to second-line	Beta	0.10	[55]
Second-line to third-line	Beta	0.01	[56]

### Model based economic evaluation

#### Semi-Markov model development

Data capture and analysis was conducted in Microsoft Excel® (Version 2010) (Microsoft Corp., Redmond, WA). Ersatz version 1.2 (www.epigear.com), a boot-strap add-in application for Excel, was used to perform the uncertainty analysis.

**Fig. 1:**
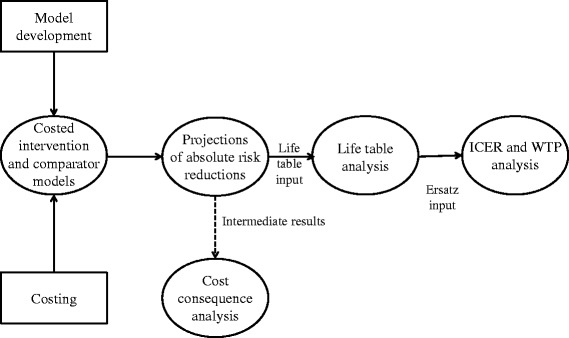
Model depicting the semi-Markov model of the HIV vaccination strategy. Healthy vaccinated and unvaccinated individuals may enter into a HIV positive state. They can progress from a HIV infection state to the HIV treatment pool. All states may progress to a death states at a rate specific to the state they were currently in

The simulation ran a semi-Markov simulation with annual cycles (Fig. 1). Tunnel states could be added to the semi-Markov model that countered the ‘memoryless’ nature inherent in the models. The vaccine was offered on a voluntary basis to adolescents from the age of ten years. The model comprised eight health states. All individuals were considered HIV negative and healthy at the start of the model (State 1). The coverage rate determined who moved into a vaccinated (State 2) or unvaccinated (State 3) state. All individuals may transition into an asymptomatic HIV state (State 4). Individuals who seroconverted to HIV positive were started on ART when eligible. Asymptomatic individuals may progress to a symptomatic (State 5) or AIDS (State 6) state. Every HIV infected individual may enter the treatment pool (State 7) which was sub-classified as 1st, 2nd and 3rd line ART regimens. Every aforementioned health state may transition to death (State 8). Each cycle carries a probability of remaining in the current health state or transitioning to another with the arrows representing the transition probabilities from one state to another. Once the vaccine had been stopped, event rates were assumed to be the same for both arms of the study.

One-way sensitivity analyses evaluated the impact of single assumptions on cost and outcomes. Probabilistic sensitivity analysis (PSA) with a bootstrapping technique of 1000 iterations was used to explore the uncertainty in the model and evaluate the robustness of the results. These results were presented as cost-effectiveness scatter plots and cost-effectiveness acceptability curves. The PSA data generated was used to determine if the intervention fell below the willingness-to-pay (WTP) threshold. As South Africa does not have a pre-defined WTP threshold, the Gross Domestic Product (GDP) per capita (2012) was used as a proxy in accordance with the WHO Guide to Cost-Effective Analysis [19, 27]. The WTP threshold was thus defined as US$ 7 508 (ZAR 61 641) per quality adjusted life year (QALY) gained. The GDP per capita range was adapted from the ‘value of statistical life’ literature and is theoretically the value of an additional healthy life year [28]. It is used in the context of this study against LYG (rather than the convention QALY) as there is no other alternative available to indicate cost-effectiveness in South Africa.

#### Cost and cost-effectiveness of a national HIV vaccination program

The programmatic costs and health implications of a vaccine implemented at US$ 12 per dose and 60 % coverage was determined and this was considered the base case. Using PSA techniques, we were able to estimate the change in cost per capita, the approximate cost per LYG and finally the cost per death averted at different vaccines prices per dose. The change from the base cost for the program cost was compared with the baseline vaccine implementation at US$ 12 per dose.

#### Life table analysis

A multi-state life table approach was used to describe the differential morbidity and mortality of a population under two alternative interventions [29]. The alternatives were the reference population displaying the HIV associated mortality experienced by the South African adolescent population under the comparator model compared with the outcomes from the adolescent population when exposed to the intervention (which was the vaccine strategy in addition to the comparator model). Disease related mortality was referenced from the literature (Table 3). The study used a cohort life table methodology which calculated the probability of death of a generation (cohort) over the course of their lifetime. Cohort life tables use age-specific mortality rates related to specific cohorts which allow for known and projected changes in mortality [30]. Within a standard life table, the disease related mortality was separated from national mortality (as shown in Eq. 1):

1 $$ {M}_{tot} = {M}_{dis} + {M}_{other} $$

Where *M*_*tot*_ is the total mortality identified in the age/sex group, *M*_*dis*_ is the *mortality* attributed to the disease state and *M*_*other*_ is the *mortality* attributed to all other causes.

The prevalence estimates for HIV was obtained from South African National HIV Prevalence, Incidence and Behaviour Survey, 2012 [2]. The ratio between the comparator and the intervention groups was used to calculate the relative reduction in HIV related mortality attributable to the intervention (reflected in Eq. 2). This reduction was applied in the life table allowing for comparisons to be made including the life expectancy, individuals surviving and the cumulative years lived.

2 $$ R{R}_m = \frac{M_i}{M_c} $$

*Where RR*_*m*_ is the mortality risk reduction, *M*_*i*_ is the mortality risk in the intervention group and *M*_*c*_ is the mortality risk in the comparator group.

Values were entered into a life table to estimate the impact of the intervention on life expectancy and the number of life years gained. Generally, a life table estimates the mortality experience of a population and calculates the life expectancy from birth [31]. The life expectancy calculated from a life table is represented by the following formula (Eq. 3) [32]:

3 $$ {e}_x=\frac{T_x}{I_x} $$

Where *e*_*x*_ is the life expectancy at age X, *T*_*x*_ is the cumulative person years lived after age X and *l*_*x*_ are the individuals alive at beginning of age X.

The difference in cumulative years lived between the intervention and comparator groups were used in the incremental cost-effectiveness ratios (ICER) calculations. The ICER represents the difference in costs between strategies and the difference in effects (e.g. LYG) between strategies (Eq. 4). The unit of measurement of the ICER is US$ per LYG gained.

4 $$ ICER=\frac{C_2 - {C}_1}{E_2 - {E}_1} = \frac{\Delta C}{\Delta E} $$

Where C_1_ and E_1_ are the costs and effects of the *standard of care* (comparator), and C_2_ and E_2_ are the costs and effects of the *intervention*.

#### Years of potential life lost

The years of potential life lost (YPLL) is used to measure the incidence of ‘premature’ mortality that occurs within a population to an age at which the death is considered untimely [33, 34]. The YPLL concept quantifies social and economic loss as a result of premature death, and has been useful in assessing specific causes of death targeting younger age groups [35]. The principle of YPLL incorporates the age at death, and the calculation is able to mathematically weight the total deaths by applying values to death at each age (Eq. 5) [34–36].

5 $$ YPLL=\varSigma \left({}_n{{\mathrm{d}}^i}_x\right) \times \left[70\hbox{--} \left(n \times 5\right)\right] $$

Where _*n*_d^*i*^_*x*_ is the number of deaths due to HIV/AIDS from age x to age x + n and n is the width of the age interval (in this study ten-year age intervals were used) and *5* represents the number of years till the midpoint of the age interval is reached.

#### Cost consequence analysis

The absolute risk reduction (ARR) was then measured as a percentage. This represented the change in the risk of an outcome of the intervention in comparison to the comparator. It was calculated as the difference in the mean values of the parameter of interest and an example of the calculation is shown in Eq. 6.

6 $$ \mathrm{H}\mathrm{I}\mathrm{V}\ {\mathrm{incidence}}_{\mathrm{comparator}}\hbox{--}\ \mathrm{H}\mathrm{I}\mathrm{V}\ {\mathrm{incidence}}_{\mathrm{intervention}} = \mathrm{A}\mathrm{R}\mathrm{R}\ \left[\%\right] $$

Where the HIV incidence _comparator_ and HIV incidence _intervention_ represented mean percentages and the difference in values was the absolute risk reduction percentage.

The difference in per capita costs with and without the intervention was then divided by the ARR values obtained for HIV incidence and HIV mortality to yield the cost per percentage reduction in disease. The outcomes for both the ARR and the per percentage reduction in disease burden was described by gender to highlight the areas of greatest impact.

#### Model assumptions

All participants entering the model were considered sexually naïve. Drop-out rates were not accounted for as all children of school-going age were assumed to be attending school. The model assumed that the rollout and uptake of HIV counselling and testing (HCT) strategies and the national rollout of the HIV vaccination strategy occurred within the school-based health services that provided comprehensive care to all socio-economic levels of learners. Finally, the model assumed good uptake of school-based health services given the provision of care in a familiar and safe environment with no encroachment on school attendance. As no formal pilot studies have been reported, there remains no validation of this assumption.

### Ethical consideration

Ethical approval for the study was obtained from the Human Research Ethics Committee (Medical) of the University of the Witwatersrand.

## Results

### Costs of models

The annual per capita cost of the comparator was US$ 80. Annual HIV vaccination per capita cost was calculated at US$ 89, representing an 11 % increase in costs. Table 4 describes the complete breakdown of these costs. There is no appreciable difference in human resources and laboratory costs associated with the vaccine intervention, though the intervention does represent a saving on both these costs. However, the intervention does predict an increase (31 %) in pharmaceutical costs driven by the need for vaccine boosters to attain durable protection. The vaccine price considered in Table 4 was US$ 12.

**Table 4:** Model components and cost comparison of the HIV vaccination program (US$). Complete breakdown of costs relating to the intervention and the comparator. The intervention comprises both the vaccine strategy and the comparator costs

Cost category	Per capita expenditure			
	Intervention	Comparator	Difference	(% change)
Laboratory	12.73	13.09	0.35	(-2.78)
HIV rapid testing	1.06	1.41		
CD4 count	4.05	4.05		
Pap smear	1.13	1.13		
Viral load	5.95	5.95		
Creatinine	0.53	0.53		
Pharmaceuticals	39.86	30.21	9.64	(+31.92)
STI treatment	1.11	0.92		
Condom distribution	1.35	1.12		
Contraception	0.77	0.63		
Anti-retroviral therapy	27.34	27.34		
Vaccine	8.94	0.00		
Vaccine delivery^a^	0.15	0.00		
Bactrim® prophylaxis	0.20	0.20		
Human resources	35.94	36.67	0.63	(-1.76)
PHC nurse	20.70	22.24		
Counsellor	11.03	11.81		
Enrolled nursing assistant	1.68	0.00		
Medical officer	0.46	0.46		
Medical specialist	2.07	2.07		
Transport ^b^	0.47	0.47	-	-
Total	89.00	80.34		

^a^Vaccine delivery includes the needle, syringe and alcohol swab for administration

^b^Calculated from average car rental cost incurred in providing a school-based service

### Uncertainty analysis

#### The cost and cost-effectiveness of a national HIV vaccination program

Implementing a South African national HIV vaccination program at the base vaccine cost of US$12 per dose (Table 5) would be considered cost-effective at US$ 5 per LYG. When benchmarking this against the WHO cost-effectiveness criteria (US$ 7508 per QALY gained), a HIV vaccine at US$ 12 is deemed highly cost-effective. However, introduction of the HIV vaccine at considerably reduced price per dose will significantly impact the future sustainability of the program. At the low vaccine cost of US$ 6, the program cost will be reduced by 5 % (US$ 52 million) of the base vaccination program; and will result in an ICER of US$ 2 per LYG. The very low vaccine price of US$ 2 would yield even better results – an ICER of US$ 1 per LYG with a 9 % reduction (US$ 84 million) in the program costs compared with the baseline vaccination strategy.

**Table 5:** Cost –effectiveness of a national HIV vaccination program at varied vaccine prices, 2012. The programmatic cost implications of varying the vaccine cost per dose were examined. The cost values reflect annual expenditure. At baseline (shaded), a vaccine at the cost of US$ 12 per dose would result in an annual cost of approximately US$ 1017 million. This represents a US$ 9 increase from the base cost per capita (Table 4). All other values have been calculated relative to the base vaccination strategy

Vaccine pricing		Program cost (millions)			Cost per		
Structure	Per dose	Total	Change from base (%)		Capita^a^	LYG	Death averted
Very low	2	933	-84	(-9)	1	1	421
Low	6	967	-50	(-5)	4	2	1106
Base cost	12	1017	-	-	9	4	2131
Medium	18	1067	50	(+5)	13	6	3161
High	24	1118	101	(+10)	18	8	4189

^a^ increase in cost per capita

#### Impact of coverage on cost and life expectancy

Table 6 explores the impact of initial vaccine and subsequent booster coverage on cost and life expectancy. To vary the coverage annually would be computationally challenging, hence the combination of the initial vaccine and the administration of the annual booster was considered as a continuum i.e. if the initial vaccine coverage was 40 %, then the booster coverage considered was also 40 %. Increasing the vaccine coverage would result in significantly increased financial investment. However increasing coverage also translated to improved life expectancy. The increased cost has to be weighed against the improved health outcomes, before the strategy is deemed cost-effective. There would also have to be consideration of the impact of other vaccine characteristics.

**Table 6:** One-way sensitivity analysis of coverage on health outcomes. By varying the coverage rates, we are able to demonstrate how an increased number of doses drive the intervention costs up

	Cost (million US$)			
Coverage	Comparator	Intervention	Increase in cost	Life expectancy
40 %	913	982	70	54.6 years
60 %	913	1017	104	55.5 years
70 %	913	1034	122	55.9 years

### Probabilistic sensitivity analysis

#### ICER and WTP results

**Fig. 2:**
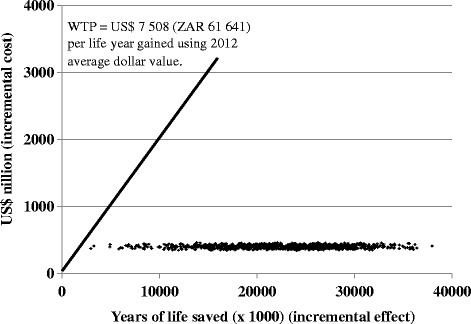
Willingness-to-pay analysis explored by varying vaccine efficacy. This figure shows the scatter plot of the costs and health outcomes from the probabilistic sensitivity analysis. The incremental cost is the difference in costs between the current treatment program and the vaccine program. Similarly, the incremental effect reflects the difference in health outcomes between the vaccine program and the current treatment program. The health outcomes are measured in years of life saved

The uncertainty around the ICER was assessed using probabilistic sensitivity analysis. The HIV vaccine intervention yielded an ICER of US$ 4.98 per LYG (95 % CI ZAR 2.77–11.61). National projections of the intervention programme were estimated to cost US$ 1017 million annually. This represents a US$ 104 million (11 %) increase on the comparator cost of US$ 913 million. Aside from the need for boosters driving the cost, it should be borne in mind that the vaccine is anticipated to reach approximately 6 million HIV negative 10–19 year old adolescents compared with the comparator strategy providing ART to 78 126 adolescents of the same age group. The intervention, however, would translate to a mean cumulative gain of 23.6 million LYG (95 % CI 8.48–34.3 million years) in the population. Apart from demonstrating the cost-effectiveness of the vaccine intervention, Fig. 2 was designed to evaluate the impact of differing vaccine efficacies on the ICER. At a vaccine efficacy of 30 %, the iterations lie on either side of the WTP threshold indicating that the intervention may not be cost-effective. However, at the vaccine efficacy of 50 % and 70 %, most iterations were considerably below the GDP per capita of South Africa, 2012. Based on this GDP, the intervention would be considered below the WTP threshold defined by the World Health Organization (WHO) and thus deemed to be highly cost-effective [19].

#### Life expectancy and potential years of life lost

The simulation of the life table results are presented in Table 7. Application of the intervention in the 10–19 year age group resulted in a 2.5 year increase in life expectancy, as well as a significant increase in cumulative gain of years lived in the age group. Importantly, as a result of the increase in life expectancy noted in the 10–19 year group, there was a reported increase documented in the subsequent age groups. The PYLL from HIV/AIDS contributing to ‘premature’ death is also given in Table 7. It is here that the impact of (the vaccine is demonstrated as there is a years of life lost without the vaccine (70 640) is considerably higher than the years lost with the vaccine intervention (48 400).

**Table 7:** Life table analysis and YPLL for 10-19 year age group

Age (x)	No vaccination (comparator)			Vaccination (intervention)		
Life expectancy	I_x_	T_x_	e_x_	I_x_	T_x_	e_x_
	(millions)	(millions)		(millions)	(millions)	
10–19	10.0	529.1	53.0	10.0	553.6	55.5
20–29	9.8	429.9	43.7	9.9	454.4	46.1
30–39	9.3	334.4	36.1	9.4	358.2	38.2
40–49	8.3	246.8	29.9	8.6	268.3	31.2
50–59	7.1	170.0	23.9	7.6	187.2	24.6
60+	5.8	105.5	18.2	6.3	117.4	18.6
YPLL						
10–19		70 640			48 400	

The movement of the vaccinated population aged 10 – 19 years of age is tracked through the life table. Columns I_x_ describes the impact of the intervention in terms of mortality reduction, columns T_x_ reflects the combined years lived with and without the intervention and columns e_x_ reflect the increase in life expectancy attributable to the intervention

I_x_ – individuals surviving, T_x_ – cumulative years lived, e_x_ – remaining life expectancy at age x

#### Cost consequence results

The 10 year absolute risk reductions in HIV associated mortality and incidence potentially offered by the HIV vaccine intervention was projected using data modelled. Table 8 described a detailed breakdown of costs to highlight the differences in vaccine impact between the genders. While all scenarios reflected an improvement in HIV related health outcome, the reduction in HIV incidence among females was notable (0.53 %), particularly given their high burden of disease.

**Table 8:** Disease risk reduction and cost consequences. The absolute risk reduction was estimated over a 10 year period

	10 year risk: mean % (SE)				Absolute risk	Cost
	Intervention		Comparator		reduction	consequence^a^
Total						
Incidence	1.08	(0.08)	1.49	(0.15)	0.42 %	20.87
Mortality	1.05	(0.01)	1.45	(0.04)	0.41 %	21.36
Male						
Incidence	1.09	(0.09)	1.51	(0.15)	0.42 %	20.67
Mortality	1.10	(0.02)	1.52	(0.05)	0.42 %	20.45
Female						
Incidence	1.40	(0.12)	1.94	(0.22)	0.53 %	16.29
Mortality	1.03	(0.02)	1.42	(0.04)	0.39 %	22.05

*HIV* human immunodeficiency virus; *SE* standard error

^a^per 1 % reduction in risk

## Discussion

The study aimed to assess the cost-effectiveness of national rollout of the hypothetical HIV vaccine to school-based adolescents. The South African HIV epidemic is widely acknowledged to be generalized, with adolescents and young adults disproportionately at risk for HIV [37]. In 2013, South Africa reported 16 % of the global HIV incidence despite concerted efforts at the national level ranging from increasing ART distribution by 75 % between 2009 and 2011 to boasting the largest and most established condom distribution program in the world [2, 38]. This earmarked adolescents as a key population to be reached if HIV prevention strategies are to impact incidence and if HIV mortality rates are to be significantly curtailed [37]. While the introduction of a potential HIV vaccine in schools represents a significant financial investment, the health outcomes in terms of improved life expectancy, markedly decreased potential years of life lost and decreases in HIV mortality and incidence are substantive. Life expectancy was equally influenced by vaccine coverage rates, while the assessment of cost-effectiveness was found to be sensitive to the vaccine efficacy.

The life table findings together with the conventionally accepted thresholds for cost-effectiveness being met demonstrate the financial plausibility of HIV vaccine implementation [19]. Importantly, the vaccine remained cost-effective even at higher prices per dose examined but at substantially greater programmatic costs. Annual HIV vaccination represents a substantial increase in costs per capita at base coverage of 60 % of HIV negative adolescents. This constitutes a significant investment considering the intense competition of several competing burdens of disease on a constrained South African health budget [39]. As much as the long term financial sustainability of the burgeoning ART program has been brought into question, the implementation of a HIV vaccine program over several decades may prove equally daunting. It is important to bear in mind that the comparator cost reflects those currently on treatment (excluding the treatment shortfall of approximately 58 % [1]) and thus represents a gross underestimation of what we should be paying if those unable to access treatment were indeed able to access it. Another major consideration is that the upscaling of ART may not impact the HIV incidence as definitively as a primary preventative strategy may. It must be remembered that while averting infections has a cost attached from a government perspective, it may also give rise to the substantial financial gains of reducing the demand for ART [40].

South Africa has successfully negotiated reduced pricing for ART and HPV vaccines in the past, and this bodes well for future procurement of HIV vaccines [23, 24], as the price is undetermined at this point. If vaccine development fails to reduce the number of annual boosters required to maintain protection, then the pricing represents a key factor in deciding the cost-effectiveness of the intervention. Apart from the economic impact, HIV vaccine implementation has the capacity to influence long term health outcomes. The mean cumulative gain of LYG could support efforts to improve life expectancy in the country, an area identified as a strategic output of the National Service Delivery Agreement [5].

The South African epidemic is predominantly heterosexual. This work represents an over-simplification of the rather complex sexual networking structures at play in the South African HIV epidemic. Nonetheless, those individuals at high risk may still acquire infection ascribed to repeated risk exposures despite the protection conferred by the vaccine compared with those at low risk. At a population level, the premise remains that a partially effective vaccine may still avert or delay infection even if it is unable to completely prevent an infection from establishing [41]. Assessment of a partially effective vaccine in the United States of America (USA) emphasizes that even modest and temporal reductions in HIV infections have important benefits at the population level [42]. Andersson et al demonstrated similar health benefits to the USA study when modelling the RV144/Thai trial vaccine in South Africa, but cautioned that a vaccine of limited duration could only be effective with high coverage levels, which translated to millions of doses [43].

Adolescents are a critical target for this intervention. Apart from being a key population identified in the transmission of HIV, adolescents in a school environment appear more easily accessible as a target group considering that more commonly identified high risk groups such as commercial sex workers are often harder to reach due to stigma and marginalization [43]. However, adolescents have historically encountered barriers in trying to access health services in South Africa from confidentiality issues to the judgmental attitudes of staff. It is not surprising that they often do not return for follow up care [9]. The school environment could be deemed a “safe space” for peer discussion and accessibility of relevant health services. Neglecting the comprehensive health needs and barriers to care of this adolescent population has the potential to undermine the success of HIV prevention initiatives [44]. Further, low social acceptability of HIV vaccines fueled by the fear of vaccines and poor side effect profiles present potential deterrents to uptake and coverage [45]. It is understandably difficult for hypothetical scenarios to emulate real-life behavioral changes but knowledge of these factors underscores the need for comprehensive sexual education and risk reduction counselling; which could prove more plausible in the school environment [46].

This study had several limitations. Firstly, it is unclear to what degree behavioral disinhibition may occur following vaccination as this was not assessed in the model. Changes in sexual risk behavior post HIV vaccination are poorly understood in the African setting [46]. In high HIV prevalence communities like South Africa, a decrease in condom use even with stable partners would likely result in an increase in HIV rates [46]. In fact, South African data has inferred that poor comprehension of the ‘low-efficacy’ concept was associated with a reported potential decrease in condom use. It is further postulated that the degree of behavioral disinhibition may depend largely on the manner in which the vaccine effects are marketed to the public and vaccine recipients alike [40]. The impact of risk compensation becomes critical when considering the low efficacy displayed by the candidate vaccines thus far [46]. Secondly, the study was unable to assess the effects of herd immunity. Notably, Long et al. alluded to partial efficacy vaccines providing some benefits to the unvaccinated population through herd immunity [42]. This is particularly important considering the low coverage rates of childhood vaccinations in South Africa as it speaks directly to the country’s capacity to introduce and implement a HIV vaccine [22]. At 60 % coverage, this program calls for an unprecedented 5.9 million adolescents to be vaccinated. Given this, it is not surprising that implementation costs are high. Thirdly, the provider perspective was considered as the largest burden of direct medical program costs will be borne by the healthcare sector. Although the societal costs were not analyzed, its contribution would be substantial and could improve the overall cost-effectiveness of the vaccine. Fourthly, booster vaccinations were not assessed in the original RV144/Thai trial work [42]. Therefore the assumption that booster vaccination would provide the same protective effects as the initial vaccination was hypothetical. There has been limited description of this in the literature [46]. Additionally, administration costs would drastically increase the program costs with the need for annual boosters. This is the key cost factor implicated in the difference between the comparator and intervention cost. However, it is hoped that attrition rates of vaccine recipients would be minimized by targeting the relatively stable school population. Lastly, this study has considered HIV vaccination as an isolated intervention apart from the ART rollout and condom distribution. In the clinical setting, this intervention would probably work synergistically with other prevention strategies such as male medical circumcision and an optimal combination of strategies should be better defined once data becomes available [40, 42]. As discussed earlier, the limited success achieved in curbing the national HIV incidence by the current public sector HIV prevention strategies warrants the evaluation of strategies on its individual merits.

## Conclusion

In conclusion, these findings suggests that a national HIV vaccine program administered to adolescents in South Africa would be a cost-effective means for reducing the massive disease and economic burden of HIV. The implications on health outcomes are significant with reductions in HIV associated mortality and incidence and improved life expectancy demonstrated by the model. However, a vaccine with more durable protection and requiring fewer boosters would considerably reduce costs. While this work provides decision makers with objective baseline data for considering the adoption of the potential HIV vaccination intervention nationally, more realistic estimates on cost and disease burden should be gauged once the efficacy, duration of protection and vaccine cost is determined.

## Abbreviations

CI: confidence interval
GDP: Gross Domestic Product
ART: highly active antiretroviral therapy
HIV: Human Immunodeficiency Virus
ICER: incremental cost-effectiveness ratio
PHC: primary healthcare
PSA: probabilistic sensitivity analysis
QALY: quality adjusted life year
UPFS: uniform patient fee schedule
USA: United States of America
US$: United States Dollar
ZAR: South African Rand
